# Bridging global paleoscientists: an interview with PAGES Executive Director Marie-France Loutre

**DOI:** 10.1093/nsr/nwaf260

**Published:** 2025-06-27

**Authors:** Weijie Zhao, Yan Zhao

## Abstract

The Past Global Changes (PAGES) project was established in 1991, initially as one of the core research projects under the International Geosphere-Biosphere Programme (IGBP) of the International Council of Scientific Unions (ICSU). PAGES is an international scientific organization that connects paleoscientists worldwide. Over 5000 scientists from >125 countries currently subscribe to PAGES and all interested scientists are encouraged to get involved.

In May 2025, the 7th PAGES Open Science Meeting and the 5th PAGES Young Scientists Meeting took place in Shanghai, China. The conferences focused on ‘Earth System Changes from the Past towards the Future’ and attracted ∼800 experts from >40 countries to attend and communicate. Seizing this opportunity, NSR interviewed Dr. Marie-France Loutre—executive director of PAGES and based at the International Project Office in Switzerland.

Dr. Loutre is a Belgian paleoclimate modeling specialist and has been directing the PAGES office for almost 10 years. During our interview, Dr. Loutre provided a comprehensive overview of the organizational structure and scientific initiatives of PAGES. She particularly emphasized PAGES' support for early-career researchers and its commitment to community diversity, while also sharing her reflections and insights from working in an international scientific coordination project.

## Teaser

A comprehensive overview of PAGES' organizational structure and scientific initiatives, emphasizing its support for early-career researchers and commitment to community diversity.

## THE PAGES ORGANIZATION

***NSR:*** What is PAGES?

***Loutre:*** PAGES is already >30 years old. It is a platform connecting global paleoscientists, regardless of where they come from, their age, gender, career status, etc. We do not do science per se; rather, we coordinate and promote exchange between researchers.

Our primary objective is to improve the understanding of past global changes in climate, environment, biodiversity, human impact and other aspects. By understanding what happened in the past, one can better predict what will come in the future, and also inform policymakers for strategies to make a more sustainable world.

But I should explain what the ‘past’ is for PAGES. The past that PAGES community members study starts when there is no direct observation record, so it depends on what you are looking at and where you are. For example, for temperature in Europe, direct thermometer measurements started a few hundred years ago and before that time is what is considered the past. But, for other places, for which there is no historical temperature record, the past starts yesterday and can be traced backwards to the beginnings of Earth. In practice, for PAGES, the past goes from the recent past until a couple of million years ago. We usually do not go much further back than that—until now, we have not dealt with the dinosaurs.

***NSR:*** How is PAGES organized?

***Loutre:*** PAGES is funded from two sources, equally: the Swiss Academy of Sciences (SCNAT) and the Chinese Academy of Sciences (CAS). PAGES is also a Global Research Network of Future Earth, which supports PAGES with a small amount each year.

**Figure 1:**
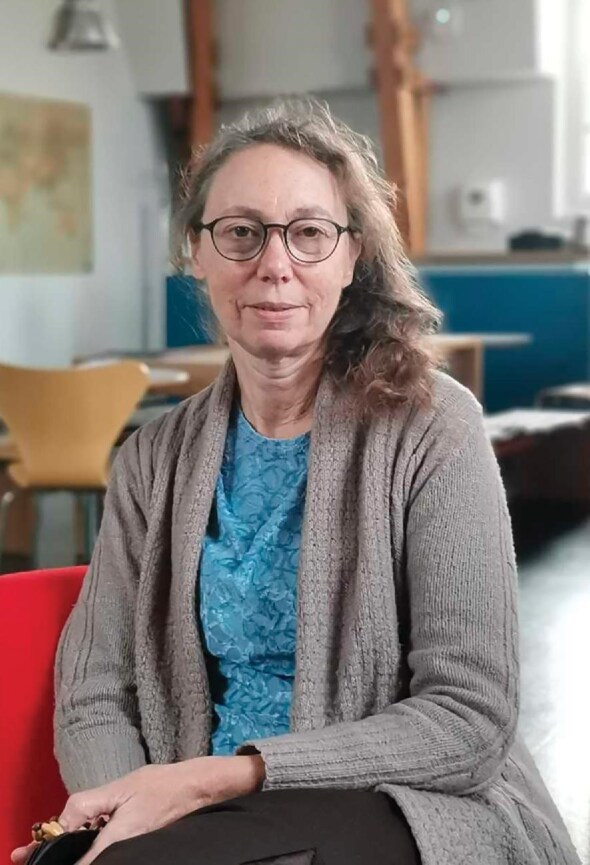
Dr. Marie-France Loutre. *(Courtesy of the interviewee)*

The International Project Office (IPO) is in Switzerland and hosted at the University of Bern. The university does not fund us directly, but it helps us a lot by providing us with offices and access to its library, as well as assisting us with all the administrative matters. The office currently has six people and most of our work is to circulate information. We edit two PAGES journals—one published twice a year and the other irregularly. We also send a newsletter to our members every month with the most recent updates—new articles that have been published, upcoming workshops, important deadlines and so on.

The PAGES project is guided by an international Scientific Steering Committee (SSC) and PAGES’ core activities take place within the working groups. We currently have 15 working groups, big and small, focusing on different topics. But, importantly, it's not the office or the SSC that decides what topics a working group should work on—it's up to the researchers to decide. If they want to tackle a specific scientific question and one group is not enough, then they can decide to join forces to work together and submit an application to set up a working group. The SSC oversees the applications and that is really what PAGES strives for: collaborative ideas from scientists all over the world.

***NSR:*** What's your expectation of PAGES in the coming 5 years?

***Loutre:*** I hope PAGES remains the same, but evolves. Several research topics set at the very beginning of PAGES are still those that we are tackling today. I think these will remain the same, but the way we work on these issues will be different and new discoveries keep emerging. Despite all the changes, it is important to keep the core of what PAGES is.

## THE SCIENCE OF PAGES

***NSR:*** Please briefly introduce the current working groups. What are they focusing on?

***Loutre:*** One working group is called the 2k Network. They published an important paper in 2019 [*Nat Geosci* 2019; **12**: 643–9], reconstructing temperature over the last 2000 years. This result was adopted by the IPCC, the Intergovernmental Panel on Climate Change. And, now, this group is working on the reconstruction of past dry and wet conditions.

There is a long-term working group, PALSEA, studying sea-level changes. Knowledge of the past can inform us of future sea levels. We can go to the past and see what the sea level was at a certain temperature, CO_2_ concentration and with other influencing factors, and use that information to predict what is expected in 50 or 100 years under the same conditions.

Some groups reconstruct the past from human archives, including journals, manuscripts, paintings and poems. They try to extract climate or landscape information from all of those records.

Several other groups work on different types of natural archives. For example, one group named SISAL is working on speleothems in caves to reconstruct past climates.

That is really what PAGES strives for: collaborative ideas from scientists all over the world.

—Marie-France Loutre

Then there are groups that are more involved in the human aspect, studying interactions of the environment and human society. One famous question is: How and why did some of the once flourishing societies suddenly disappear? Is it climate or environmentally related? And it's interesting to study whether the disappearances of several remote societies are simultaneous or not. Another question is: To what extent are the environmental changes that we observe caused by human activities versus natural variability? The answer to this question is important because, if you want to restore a natural environment, such as that in Madagascar, then you need to set the restoration point. If the reduction of forests actually started before human impact and is a natural process, then it would be a question of whether we should plant the trees back or not.

***NSR:*** Will there be new working groups coming soon?

***Loutre:*** We have been approached by six or seven people who are interested in submitting an application for a new working group. As PAGES has a limited amount of money, the number of groups cannot grow too much, so the turnover of groups is important to make space for new ones. Our working groups are usually set for a maximum of two 3-year phases, and we encourage them to set goals that are reachable in 3–6 years. It is preferred that specific goals are set, and subsequently met within this period, with scientifically sound papers published. And, after that, the scientists may become interested in other topics and be involved in new groups. In some cases, scientists will continue to work together under the umbrella of PAGES, but no longer as a working group. From time to time, they send us their updates and we are happy to share their results.

***NSR:*** What scientific questions about past global changes interest you personally the most?

***Loutre:*** I have been working at PAGES for almost 10 years but, before that, I was a researcher working mostly on modeling how changes in Earth's orbital parameters influenced climate in the past. So the question that currently interests me the most is the climate-change mechanisms during the Mid-Pleistocene Transition (MPT). It's very likely that new results on this question will appear in the coming years because, amongst many other research projects on the topic, a very long ice core has been retrieved from Antarctica. The ice core is almost 3000 meters long and is believed to conserve a continuous CO_2_-concentration record for >1.2 million years—much longer than the previous record of 0.8 million years. That may bring exciting new discoveries.

***NSR:*** In which research areas do you hope Chinese researchers will be actively involved?

***Loutre:*** Chinese scientists are famous for studying the loess, which is related to dust in the atmosphere. There are still questions to be answered on its impact on climate, so that's probably one aspect. Other topics that Chinese scientists may make contributions to include monsoon, speleothem, the deep ocean and others.

There are some strong and large research groups in China, and sometimes it seems that they can survive on their own and do not need to collaborate with anyone outside of China. But communication is really important for science and I hope that PAGES will act as a platform for Chinese scientists to communicate with the international world. I hope that Chinese scientists will become more actively engaged in PAGES working groups and activities.

**Figure 2:**
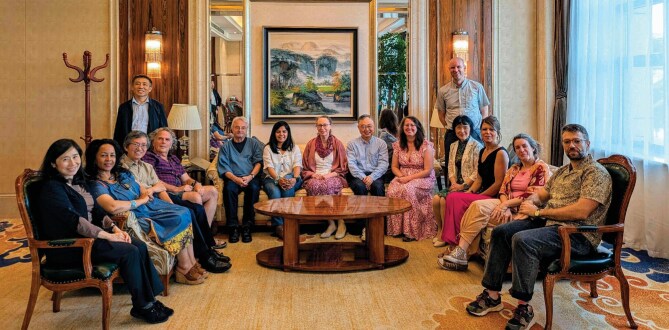
PAGES Scientific Steering Committee 2025. Photo taken from day one of the SSC Meeting held in Shanghai (China) on 26 May 2025. Left to right: Ayako Abe-Ouchi, Aster Gebrekirstos, Aixue Hu, Yair Rosenthal, Shiling Yang, Martin Grosjean, Aditi K. Dave, Marie-France Loutre (PAGES IPO), Liping Zhou, Keely Mills, Fabrice Lambert, Yan Zhao, Ilham Bouimetarhan, M. Eugenia Ferrero, Iván Hernández-Almeida (PAGES IPO). Missing in the picture: Pradeep Srivastava, Lukas Jonkers, Natasha Barlow. Photo credit: PAGES.

## BRING IN YOUNG SCIENTISTS

***NSR:*** The 2025 Open Science Meeting (OSM) and associated Young Scientists Meeting (YSM) were held in May in Shanghai. What were the highlights of these events?

***Loutre:*** The OSM and YSM are the landmark events of PAGES. This is where PAGES gets most of its visibility. People meet there, exchange their most recent results and talk about new collaboration opportunities. I'm particularly happy to see interconnections between different working groups.

PAGES was one of the first to initiate a YSM—a meeting that takes place before the major event and is dedicated to young scientists. Of course, it is about science, but it is also, mostly, about building networks and improving all the skills that a scientist needs.

For some of the younger attendees, it may be their first big international conference. They may have known a couple of people before and, through these people, are introduced to more, and so on. I myself met some of my collaborators and friends at conferences when I was starting out. A person's network starts being built when one is young.

The YSM is also a great opportunity for young scientists to communicate and learn about all the skills they need as a scientist, including how to prepare a project, how to write a paper and how to cope with potential challenges.

In this Shanghai meeting, one young scientist explained that she had submitted 10–15 research project proposals, but only 2 were accepted. That is life in science, but many young scientists don't know that, and may feel frustrated when rejected. YSMs can reassure them that rejection doesn't necessarily reflect their scientific ability and encourage them to persist.

Our conferences also provide young female scientists with an opportunity to talk with their senior colleagues about how to continue their career if they wish to start a family. These questions are really important, but often young scientists are not able to ask their supervisors directly.

***NSR:*** What other support does PAGES provide for early-career researchers?

***Loutre:*** We always try to attract young scientists because they are the scientists of tomorrow and, if they are not included in our community now, then they never will be.

We have an Early Career Network within PAGES that is organized with and by the early-career researchers themselves; they have their own activities. We also financially support young scientists, often from less-favored countries, to attend workshops. For a couple of years, we ran a program to support young scientists from South America or Africa to move within their own continent for short-term visits to another lab, with the idea that it could foster future collaborations.

***NSR:*** Do you have advice for young researchers?

***Loutre:*** Don't be discouraged by rejection. Don't be afraid of discussing your papers with more senior people. And don't hesitate to follow up on an opportunity that suddenly opens up to you, even if it is not the path that you initially thought you would take. It's good to have a goal in life, but sometimes it's important that you revise that goal and, if you have to do so, don't consider that a failure. I don't think this advice is specifically for paleoscientists, but general advice for everybody.

A person's network starts being built when one is young.

—Marie-France Loutre

## KEEP AND IMPROVE DIVERSITY

***NSR:*** How does PAGES promote geographic and gender equity in science?

***Loutre:*** While PAGES does not actively promote geographic and gender equity in science itself (because PAGES does not conduct research at the IPO), we do actively voice to the different working groups that, when they have an activity, they must not forget diversity in every aspect, including gender and geography. When we discuss applications for new SSC members, we also take diversity into account. We have a code of conduct.

For female scientists, another challenge is that there are currently not many women in the field. When organizing activities, we often rely on the same few female individuals because the pool is small. It's important to bring more young women into our community to obtain real gender equality.

***NSR:*** Any advice for young female scientists?

***Loutre:*** I remember when I was young and the only woman in my lab; two secretaries were women, but I was the only female researcher. Once, in a meeting, my adviser asked me very nicely: ‘Marie-France, could you please serve us the coffee?’ Why me? Because I was a woman, of course. I don't believe he even realized what he was asking. It was just the way it is. Being a woman in science has long been a challenge.

I think we need a new generation, one more committed to equality, to make real changes. And, if you are already a little bit further in your career, try to be a role model for younger women, to show them what is possible. That's definitely not easy, but we should try.

## BE INTERNATIONAL

***NSR:*** During Trump's second term, the USA further reduced investments in climate-change research—is this affecting PAGES?

***Loutre:*** There is no direct impact because our funding has not come from the USA since 2018. But there are indirect impacts. For example, some US-based colleagues were unable to attend the Shanghai OSM because they did not have access to funding for travel anymore. More generally, if some US projects are terminated abruptly, we will lose the US researchers who are involved, and maybe also data from the USA. PAGES is a network and we don't want to see any part of the network go missing.

***NSR:*** Based on your 10-year experience in PAGES, what are the common challenges in advancing international programs?

***Loutre:*** I think being international is a challenge because the objectives of doing science are slightly different in each country and an international body needs to cope with all these different aspects. For example, in Africa, the major objective of science is to solve the problems of today—to provide food for their people. So, all the projects there, focusing on floods or drought, would ultimately go in that direction. In other places, the key issue may be energy production or something else. Resources for science are also diverse in different countries—some have more readily available resources and others much less.

In recent times, there have been more and more online meetings. Technology is making communication more convenient, but it also brings new challenges for international meeting organizations because, when we need to gather people on the west coast of the USA, in Europe, in Japan and maybe in New Zealand, it crosses 14 time zones and it's almost impossible to set a time that is comfortable for everybody. It's not directly related to science, but it's a real challenge for international science collaboration.

***NSR:*** What has been the most memorable project you’ve led at PAGES?

***Loutre:*** That is a difficult question. I think organizing the online YSM during the COVID-19 period was a challenge, but it was also memorable because it's something that we had never done before, and probably never will do again.

Apart for the time-zone challenge, it's also very difficult for participants to sit in front of a computer for half a day. One of our IT technicians helped with this issue by making the platform look a little bit like an online game. The avatars of participants could move around a nice space and actually talk to each other. At the end of the meeting, everyone found out how to make their

PAGES is a network and we don't want to see any part of the network go missing.

—Marie-France Loutre

avatars dance and all the avatars were dancing on their chairs! So, I think it was a success in the end.

It was also a time of cohesion for our office. When things weren't working at the last minute, everybody ran through the corridors and we worked together quickly, efficiently and got things done. That is a nice memory.

## Notes

Weijie Zhao is an NSR news and science editor based in Beijing; Yan Zhao is a professor at the Institute of Geographic Sciences and Natural Resources Research, Chinese Academy of Sciences, and a member of the PAGES Scientific Steering Committee.

